# Therapeutic opportunities for senolysis in cardiovascular disease

**DOI:** 10.1111/febs.16351

**Published:** 2022-01-25

**Authors:** Mark Sweeney, Stuart A. Cook, Jesús Gil

**Affiliations:** ^1^ MRC London Institute of Medical Sciences (LMS) London UK; ^2^ Institute of Clinical Sciences (ICS) Faculty of Medicine Imperial College London UK; ^3^ Wellcome Trust / National Institute of Health Research 4i Clinical Research Fellow London UK

**Keywords:** atherosclerosis, cardiovascular, heart failure, pulmonary hypertension, senescence, senolytics

## Abstract

Cellular senescence within the cardiovascular system has, until recently, been understudied and unappreciated as a factor in the development of age‐related cardiovascular diseases such as heart failure, myocardial infarction and atherosclerosis. This is in part due to challenges with defining senescence within post‐mitotic cells such as cardiomyocytes. However, recent evidence has demonstrated senescent‐like changes, including a senescence‐associated secretory phenotype (SASP), in cardiomyocytes in response to ageing and cell stress. Other replicating cells, including fibroblasts and vascular smooth muscle cells, within the cardiovascular system have also been shown to undergo senescence and contribute to disease pathogenesis. These findings coupled with the emergence of senolytic therapies, to target and eliminate senescent cells, have provided fascinating new avenues for management of several age‐related cardiovascular diseases with high prevalence. In this review, we discuss the role of senescent cells within the cardiovascular system and highlight the contribution of senescence cells to common cardiovascular diseases. We discuss the emerging role for senolytics in cardiovascular disease management while highlighting important aspects of senescence biology which must be clarified before the potential of senolytics can be fully realized.

## Graphical abstract

Cellular senescence and the senescence‐associated secretory phenotype (SASP) have increasingly been recognized as key contributors to multiple cardiovascular diseases. The emergence of senolytic therapies provide intriguing opportunities within the cardiovascular system to arrest or reverse diseases including atherosclerosis, heart failure and pulmonary hypertension. This review aims to improve the understanding of the (patho)physiological roles of senescence in the cardiovascular system to guide safe and effective use of these novel, powerful senolytic therapies.

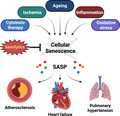

**Abbreviations**
AD: alzheimer’s disease
AMI: acute myocardial infarction
AMPK: 5’ AMP‐activated protein kinase
Bcl‐2: B‐cell lymphoma 2
CD31: cluster of differentiation 31
CDKI: cyclin‐dependent kinase inhibitors
Cdkn2a: cyclin‐dependent kinase inhibitor 2a
CPC: cardiac progenitor cells
ECM: extracellular matrix
eNOS: endothelial nitric oxide synthetase
EPC: endothelial progenitor cells
GDF15: growth/differentiation factor 15
GI: gastrointestinal
HUVEC: human umbilical vein endothelial cells
ICAM‐I: intercellular adhesion molecule 1
IL: interleukin
INK4a: inhibitor 'a' of CDK4
INK‐ATTAC: INK4a‐apoptosis through targeted activation of caspase
IPF: idiopathic pulmonary fibrosis
LDL: low‐density lipoproteins
LV: left ventricle
MDM2: mouse double minute 2 homolog
MI: myocardial infarction
MMP: matrix metalloproteinase
NAD(P)H: nicotinamide adenine dinucleotide (phosphate)
NRVCM: neonatal rat ventricular cardiomyocytes
p16‐3MR: p16 trimodality reporter
PAI‐1: proteinase inhibitor 1
pH: potential of hydrogen
PH: pulmonary hypertension
RAAS: renin–angiotensin–aldosterone system
Rb: retinoblastoma
ROS: reactive oxygen species
SASP: senescence‐associated secretory phenotype
SA‐β‐Gal: senescence‐associated β‐galactosidase
SMAD: SMA and mothers against DPP Homolog
TGFBRI: tumour growth factor β receptor inhibitor
TGF‐β: tumour growth factor β
TNF‐α: tumour necrosis factor α
TRF2: telomeric repeat‐binding factor 2
VCAM‐I: vascular cell adhesion protein 1
VSMC: vascular smooth muscle cells
αSMA: α‐Smooth muscle actin

## Introduction

Cardiovascular disease is the leading cause of mortality worldwide and the incidence increases with advancing age [1]. This progressive susceptibility to cardiovascular disease is driven in large part by the accumulation of cardiovascular risk factors throughout life and the presence of co‐morbidities which increase complications and mortality [2]. However, despite controlling for these factors, age remains an independent predictor of cardiovascular mortality [3]. One potential explanation of this phenomenon is the accumulation of senescent cells within cardiovascular organs [4]. Cellular senescence is a programmed response to cell stress which prevents the proliferation of cells with high levels of DNA damage and other macromolecular damage. Senescent cells accumulate with age and contribute to organ dysfunction. This accumulation of senescent cells is accelerated in the setting of environmental stressors provided by multiple cardiovascular risk factors [5]. Senescence is typically characterized by a permanent state of cell cycle arrest driven by the activation of the p53/p21^Cip1^ and p16^Ink4a^/Rb pathways. Senescent cells undergo many other characteristic changes: the cells increase in size, accumulate lipofuscin and develop areas of telomeric DNA damage. Senescent cells are resistant to apoptosis and develop a senescence‐associated secretory phenotype (SASP), which is pro‐inflammatory and typically targets these cells for clearance from the body by the immune system. However, if senescent cells linger, they exert a range of adverse physiological consequences which have relevance in the development of age‐related cardiovascular disease.

Impairment in autophagy, metabolic reprogramming and changes to cellular architecture can impair the ability of senescent cells to perform their primary functions leading to progressive tissue dysfunction. Moreover, senescence of progenitor cells prevents self‐renewal and limits the ability for tissue regeneration. But the SASP produced by lingering senescent cells mediates most of their pathological effects. The SASP releases high levels of pro‐inflammatory and pro‐fibrotic signalling factors which are critical to its pathologic role as the inappropriate recruitment of immune cells and deposition of excess extracellular matrix (ECM) proteins are critical components of most major cardiovascular diseases. The SASP is able to induce senescence in neighbouring cells, extending the area of tissue damage and causing further cellular dysfunction in the local environment [6].

## Main sections

### Senolytic therapies

Senolytics are an emerging drug class which are able to exploit differences between senescent and non‐senescent cells in order to selectively induce apoptosis in senescent cells. The idea was first hypothesized with the use of the INK‐ATTAC mouse in which a ‘suicide gene’ was inserted in the *Ink4a* locus (also known as *Cdkn2a*, encoding for p16^Ink4a^) which could be activated to induce apoptosis in p16^Ink4a^ expressing cells by administration of a small molecule (AP20187) [7]. Clearance of senescent cells using this system has improved health span in multiple organ systems and lifespan on an organism level [7]. Therefore, significant effort has been dedicated to identifying compounds which could selectively target senescent cells [8].

Drugs which target the anti‐apoptotic pathways upregulated in senescent cells have provided the most promise [9]. Multiple candidates have emerged which target BCL‐2 family proteins. These include ABT263 which is a specific inhibitor of BCL‐2 family leading to apoptosis induction in senescent cells [10] and more recently identified BCL‐2 inhibitors A1331852, A1155463 and UX1325 [11]. The tyrosine kinase inhibitor dasatinib has multiple receptor targets including EphrinB2 which provides anti‐apoptotic signals in senescent cells. Flavonoids such as quercetin and fisetin are naturally occurring compounds present in fruits which have multiple effects which are senolytic in certain cell types. These effects include inhibition of PI3 kinase and serpins such as PAI‐1 [11, 12]. Due to redundancy of anti‐apoptotic pathways, a combination of senolytics is needed in some cell types and the combination of dasatinib and quercetin has proven highly effective across multiple cell types and *in vivo*. These agents tend to have good side‐effect profiles with a number of the flavonoids being available in food supplements and crucially have little pro‐apoptotic effect in non‐senescent cells.

Interestingly, a commonly used cardiovascular drug, digoxin, has recently been identified to have senolytic properties [13, 14]. Digoxin is a cardiac glycoside which inhibits the Na‐K ATPase, increases the activity of the Na+/H+ exchange channel, therefore lowering the pH of the cell. Senescent cells are more susceptible to these changes than non‐senescent cells, therefore a senolytic effect emerges with this treatment [14]. Digoxin is used in the management of atrial arrhythmias and heart failure; however, its use was not associated with a mortality benefit in randomized controlled trials in heart failure [15] and subsequent meta‐analysis has suggested it may increase mortality which is likely due to the arrhythmic effects related to increased intramyocardial calcium [16]. Therefore, the use of cardiac glycosides in cardiovascular disease may not be ideal given the risk profile of these patients.

As the SASP is the predominant driver of senescence‐mediated tissue damage, therapies which reduce or modify the SASP rather than ablating senescent cells are also being actively explored. These are known as senomorphic therapies and have the potential to provide a large proportion of the positive effects of the senolytic therapies with reduced toxicity profiles which could make them more clinically acceptable. However, senomorphic therapies might not be effective in intermittent regimes.

Despite their relative novelty, senolytic therapies are already being trialled in the clinical setting in both malignant and non‐malignant disease. Outside of malignancy, a novel Bcl‐2 family inhibitor, UX1325, is being trialled in diabetic macular oedema (NCT04537884). Fisetin or dasatinib and quercetin in combination are currently ongoing investigation of the effect on skeletal muscle health in ageing (NCT04313634), osteoarthritis (NCT04770064) and Alzheimer’s disease (NCT04063124) (Table 1).

**Table 1:** Compounds with senolytic properties tested in clinical trials and common associated toxicities. AD, Alzheimer’s disease; IPF, idiopathic pulmonary fibrosis; GI, gastrointestinal.

Drug	Primary Targets	Clinical Trials to date	Clinical toxicity
ABT263 (Navitoclax)	Bcl‐2	Solid and haematological malignancy	Thrombocytopenia, neutropenia
UBX1325	Bcl‐xl	Diabetic macular oedema	None reported to date
Dasatinib	Bcr/Abl, SRC, Ephrin		Anaemia, thrombocytopenia, neutropenia, pleural effusion, GI disturbance
+ Quercetin	Bcl‐2 IGF‐1, HIF‐1α	Frailty, AD, ageing, chronic kidney disease, IPF, bone health	None reported to date
+ Fisetin	Multiple targets	Frailty, ageing, bone health	None reported to date
+ Luteolin	Multiple targets	None in senescence	Raised liver enzymes (mice)
Cardiac glycoside	Na+/K+ ATPase	None in senescence	Arrhythmia, GI disturbance, visual disturbance
UBX0101	MDM2	Osteoarthritis	None reported to date
Geldanamycin	HSP‐90	Solid and haematological malignancy	Hepatoxicity
Tanespimycin	HSP‐90	Solid and haematological malignancy	GI disturbances, fatigue
Alvespimycin	HSP‐90	Solid and haematological malignancy	Neutropenia, fatigue, Gi disturbances
AZD8055	mTOR	Solid malignancy	Hepatotoxicity

The first reports of the effects of senolytics in pulmonary fibrosis are encouraging. A single arm, open label study with an intermittent dosing schedule of dasatinib and quercetin in 14 patients did not report any serious adverse events and functional measures including 6 min walk and timed chair stands improved in patients compared to baseline. Circulating SASP factors including IL‐6 and MMP7 showed a non‐significant trend towards reduction. However, the trial was insufficiently powered to provide conclusive evidence of this and further studies will be necessary and are ongoing [17]. Initial phase II studies in patients with osteoarthritis using UBX0101, a p53/MDM2 interaction inhibitor, failed to show any improvement in pain following a single intra‐articular injection; however, no serious toxicities emerged during this trial.

Despite the cardiovascular system contributing significantly to age‐related disease burden, there are currently no clinical trials of senolytic therapies ongoing in cardiovascular disease. A major difference between management of cardiovascular disease and in particular preventive cardiovascular medicine compared to malignant disease is the limited tolerability of toxicities and side effects. This limits a number of current senolytic therapies from being viable translatable targets, in particular ABT‐263, which is associated with dose‐limiting thrombocytopenia. Preclinical studies of these pathways remain useful as alternatives with better toxicity profiles are developed. We aim to review the accumulating evidence to support targeting senescent cells within the cardiovascular systems and outline the potential obstacles which may emerge with this approach.

## Senescent cells within the cardiovascular system

Senescence has been described across the majority of cell types of the cardiovascular system including the most abundant cells: cardiomyocytes, endothelial cells and fibroblasts as well as smooth muscle cells, immune cells and cardiac progenitor cells (CPC). The drivers of senescence are similar across multiple cell types with common factors including telomeric shortening, oxidative stress, mitochondrial dysfunction and DNA damage [5]. However, given the marked difference in function, replicative capacity and differentiation stage of cells in the heart, the way in which the senescence phenotype presents can vary markedly across cell types.

### Cardiomyocytes

#### Cardiomyocyte senescence does not involve cell cycle arrest

Cardiomyocytes makeup 25–35% of cells within the adult heart [18] and constitute 65–70% of the myocardial volume [19]; however, cardiomyocytes exit the cell cycle in the early post‐natal phase, are terminally differentiated and post‐mitotic. Therefore, exit from the cell cycle cannot be used to define the senescent state of cardiomyocytes. However, there are other examples of post‐mitotic cells undergoing senescence, and other senescence‐associated features are present in cardiomyocytes exposed to a range of stimuli which would be compatible with the onset of a form of senescence. Anderson et al recently characterized a senescent phenotype in ageing mouse cardiomyocytes which included the expression of cyclin‐dependent kinase inhibitors (CDKI) p16^Ink4a^ and p21^Cip1^ and the induction of senescence associated‐β‐galactosidase (SA‐β‐gal) activity [20, 21]. Cardiomyocytes extracted from aged mouse hearts also developed a secretory phenotype which although did not match the classically described SASP, it did include a number of pro‐fibrotic factors including TGF‐β2, endothelin‐3 and Gdf15 [20]. Additional changes in aged cardiomyocytes include increased cell size which in combination with increased fibrosis led to increased left ventricular mass at an organ level.

#### *In vitro* models of cardiomyocyte physiology

The understanding of the molecular processes underlying cardiomyocyte senescence has been hampered by a relative difficulty in modelling cardiomyocyte biology *in vitro*. Neonatal rat ventricular cardiomyocytes (NRVCM) can survive in culture and cardiomyoblast cell lines such as H9c2 are not terminally differentiated and can be subcultured. However, these systems are imperfect models of senescence development in post‐mitotic adult cardiomyocytes and significant challenges exist in extrapolating results from these systems to adult cardiomyocyte physiology. Despite these limitations, multiple studies using these experimental setups have provided an indication of the factors affecting senescence within the cardiomyocyte.

#### Telomere shortening is an important factor driving cardiomyocyte senescence

Shortening and damage to telomeres within cardiomyocytes have been reported in multiple cardiomyopathy syndromes including hypertrophic cardiomyopathy and dilated cardiomyopathy as well as in the ageing heart [20, 22, 23, 24]. NRVCM, despite being unable to divide or replicate, develop characteristics of senescence after 13 days in culture and by 21 days up to 80% of cells are positive for SA‐β‐gal, p21^Cip1^ and p53. Telomerase activity is reduced in these cells, resulting in shorter telomeres, changes which are also apparent in the aged mouse hearts [25, 26]. Anderson provided evidence for the role of telomere damage as a causative factor for cardiomyocyte senescence by using telomere targeting endonucleases in H9c2 and NRVCM which cause these cells to express p21^Cip1^, induce SA‐β‐gal activity and increased cell size [20]. Progressive reduction in telomere length across generations in telomerase‐deficient mice resulted in a heart failure phenotype by generation 4. This phenotype was dependent on p53‐mediated inhibition of proliferator‐activated receptor gamma co‐activator 1‐α and 1‐β leading to impaired mitochondrial biogenesis and the emergence of a phenotype resembling dilated cardiomyopathy [27, 28].

#### Mitochondrial dysfunction and ROS production

Oxidative stress has been proposed as a driver underlying telomeric dysfunction and senescence within cardiomyocytes. Oxidative stress increases in the ageing heart and these changes are accelerated by conditions including obesity, hypertension and diabetes, which are associated with cardiovascular mortality [29]. Multiple factors contribute to the increased activity of ROS‐producing enzymes such as the NAD(P)H oxidases, reduced activity of antioxidants and increased ROS production by dysfunctional mitochondria [29]. Cardiomyocytes have a high relative proportion of mitochondria due to their high energy requirements. Therefore, they are more susceptible to accumulation of dysfunctional mitochondria. Oxidative stress has repeatedly been implicated as a pathological mechanism in the ageing heart [20, 30] and treatment with antioxidants such as N‐acetylcysteine has been shown to reduce telomeric DNA damage and senescence in cardiomyocytes [20]. Mitophagy is a cellular housekeeping process aimed to remove damaged mitochondria. Mitophagy is impaired in the ageing or diseased heart [31, 32], which contributes to persistent mitochondrial dysfunction. Restoring mitophagy by overexpressing the ubiquitin ligase Parkin, which targets damaged mitochondria for removal, prevented the development of senescence in NRVCM and improved contractile response of the heart to dobutamine *in vivo* [33].

Therefore, as a result of ageing or cell stress, cardiomyocytes enter a state with impaired functional capacity, increased cell size and multiple markers of senescence including a SASP. The emergence of this phenotype appears to be associated with organ‐level dysfunction and these cells with characteristics of senescence are amenable to currently available senolytic treatments [20, 34].

### Fibroblasts

In comparison to the paucity of evidence in cardiomyocytes, senescence has been extensively studied in fibroblasts. Senescent fibroblasts play important roles in diseases such as pulmonary fibrosis, renal fibrosis and multiple cancers [35, 36]. Fibroblasts undergo a more classical form of senescence with cell cycle arrest predominating and the development of a pro‐inflammatory SASP. During the acute phase following injury, the emergence of senescence within invading fibroblasts may have roles to limit excessive fibrosis deposition and encourage regeneration in the injured area [37]. However, despite the role of senescence in limiting collagen synthesis, a persistent pro‐inflammatory SASP develops following senescence induction in fibroblasts [38, 39]. These cells appear to enter a non‐secretory but pro‐inflammatory state. While this reduces direct ECM production from the cell, over time the chronic inflammatory state contributes to ongoing tissue damage [40].

#### Aged myofibroblasts may undergo senescence upon activation

Senescence has also been reported as a consequence of myofibroblast activation. Indeed, a high proportion of α‐SMA‐positive myofibroblasts express p16^Ink4a^ [41]. The pro‐fibrotic cytokine TGF‐β induces fibroblast proliferation and myofibroblast activation, it has also been shown to induce and reinforce a senescence phenotype within fibroblasts particularly those previously exposed to cell stress [42, 43, 44, 45]. The response to TGF‐β stimulation is defective in aged fibroblasts which exhibit lower cell motility in response to TGF‐β stimulation and impaired myofibroblast transition. Aged fibroblasts have up to 60% reduced expression of TGFBRI and a blunted canonical response through SMAD activation [46], suggesting that in response to cell injury aged fibroblast have a less brisk pro‐fibrotic response and instead develop a senescent pro‐inflammatory state which persists chronically and leads to long‐term low‐level tissue damage [46].

### Endothelial cells

#### Ageing‐associated endothelial dysfunction is associated with endothelial senescence

Endothelial cells have a crucial role in the pathogenesis of a number of high prevalence cardiovascular diseases including heart failure and atherosclerosis [47]. Ageing is known to impair endothelial function which manifests as increased arterial stiffness, impaired flow‐mediated vessel reactivity and increased intimal thickness as invading inflammatory cells and cholesterol accumulates in vessel walls [48].

Senescent cells expressing p21^Cip1^ and p53 accumulate within the vascular endothelial with increasing age – a process which is delayed by regular exercise [49, 50]. The phenotype, which develops with senescent endothelial cells in culture either following stress‐induced or replicative senescence, is analogous to this endothelial dysfunction phenotype observed at an organ level. Senescent endothelial cells alter the expression of vasoactive mediators, including nitric oxide [51, 52, 53], increase expression of pro‐fibrotic cytokines such as endothelin‐1 [54] and increase expression of pro‐inflammatory adhesion molecules including VCAM‐1 and ICAM‐1 which recruit immune cells to the vessel wall and the surrounding tissue [55].

#### Angiogenic potential is impaired by senescence

Additionally, higher passage human microvascular endothelial cells have reduced tube‐forming ability and reduced mobility in cell culture [56], which will impair intimal repair or angiogenesis [57]. Endothelial senescence is accelerated in both cell and animal models of numerous cardiometabolic diseases including diabetes, obesity and hypertension [58, 59, 60, 61]. This is concordant with the impairment in endothelial dysfunction and increased cardiovascular complications seen with these diseases and reduced myocardial capillary density associated with endothelial senescence in high‐fat diet‐treated mice [62].

Furthermore, senescent endothelial cells have been identified as a source of circulating microparticles. These vesicles of concentrated signalling molecules, cytokines and RNA are produced by budding of the plasma membrane and can act as long‐distance signalling system in the body [63]. Microparticles are released from high passage primary porcine coronary endothelial cells in culture [64] and in low shear stress conditions, as occurs at the site of atherosclerotic lesions [65]. Endothelial microparticles released from senescent cells increase reactive oxygen species production, impair nitric oxide signalling and induce senescence in early passage endothelial cells *in vitro* [64]. Clinically, serum levels of CD31+/Annexin V+ microparticles correlate with cardiovascular events [66].

### Vascular smooth muscle cells

Vascular smooth muscle cells (VSMC) form the layer of cells adjacent to the endothelium and are responsible for maintaining tone within the vessel wall. Similar to the endothelial layer, they are exposed to circulating toxins, inflammatory mediators and changes in blood pressure. VSMC extracted from hypertensive [67] or hyperglycaemic subjects [68] have reduced proliferative capacity in culture compared to those from control animals and display canonical features of senescence including SA‐β‐gal expression, telomere shortening, increased expression of p16^ink4a^, growth arrest and the development of a SASP which recruits inflammatory and immune cells to the vessel wall [69]. Changes specific to VSMC senescence include a reduced reactivity to vasoactive mediators, shifting from a contractile to a pro‐fibrotic, synthetic and pro‐inflammatory phenotype [12, 70].

### Immune cells

Immune cells of both myeloid and lymphoid lineages have multiple roles in the ageing cardiovascular system. Leukocytes and, in particular, macrophages are recruited by SASP factors and as a result accumulate in the heart following damage and with increasing age. [71]. Ineffective immune cell clearance of senescent cells is common with increasing age contributing to the accumulating senescent cells. Macrophages become senescent both in response to SASP and as a result of activation and polarization [72]. Senescence within macrophages has also been reported in response to oxidized LDL within atherosclerotic lesions and due to lipopolysaccharide administration *in vitro* [73, 74]. It has been argued that senescent macrophages may be the most abundant senescent cell in areas of tissue damage and a significant proportion of the senolysis is due to clearance of senescent macrophages [72].

### Cardiac progenitor cells

Cardiac progenitor cells (CPC) have been identified as a multi‐potent, self‐renewing population of cells present within the myocardium [75, 76]. CPC isolated from older adults (76‐ to 86‐year‐old) demonstrate shortening of telomeres compared to young‐ or middle‐aged adults and other characteristic features of senescence including p16^Ink4a^ expression, SA‐β‐gal activity and markers of DNA damage. These cells develop a pro‐inflammatory SASP in response to doxorubicin treatment and can induce paracrine senescence in neighbouring cells [77]. CPC have shown some promise as a regenerative therapy and injection of young CPC into the peri‐infarct region of the mouse heart leads to improvement in LV function. The degree to which this is caused by regeneration compared to remodelling due to paracrine signalling is debated, however, injection of senescent CPC does not improve cardiac function [77]. Additionally, following genetic or pharmacological clearance of senescent cells from the aged mouse heart, the number of CPC increases and there are increased numbers of small cardiomyocytes suggestive of some degree of regeneration [77].

## Senescence and cardiovascular diseases

### Atherosclerosis

Atherosclerosis is a prototypical age‐related disease and is responsible for a massive burden of morbidity and mortality worldwide. A degree of arterial atherosclerosis is almost universally present by the age of 50 [78] with complications resulting from this increasing markedly over the following decades. Atherosclerosis is a complex process involving multiple cell types both resident in the vessel wall and recruited to the vessel by chemotactic signals and adhesion molecules. Interplay between multiple cell types is responsible for the development of the pro‐inflammatory signalling which results in the progression from fatty streaks containing macrophages and lipids in the vessel wall to complex fibrous plaque which can obstruct flow and rupture causing acute ischaemic events [79].

#### Senescent cells accumulate at atherosclerosis sites in the vasculature and are pro‐inflammatory

**Fig. 1:**
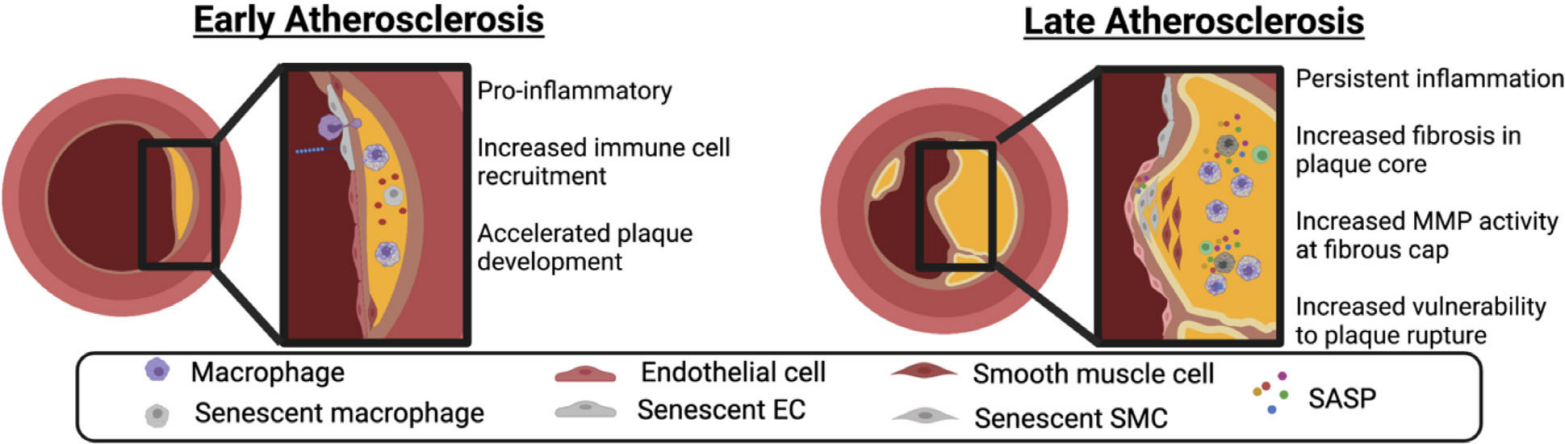
Atherosclerosis and senescence. The accumulation of senescent cells occurs early in the pathogenesis of atherosclerosis and involves multiple cell types. Endothelial cells exposed to disturbed flow at arterial branch points develop changes characteristic of senescence. These changes include the development of a pro‐inflammatory SASP and expression of vascular adhesion molecules which attract inflammatory cells to the vessel wall and accelerates the development of atherosclerotic plaques. Macrophages within the tunica media become senescent when exposed to oxidized LDL leading to further release of a pro‐fibrotic and inflammatory SASP. Senescent cells within the fibrous cap of atherosclerotic lesions produce extracellular matrix remodelling enzymes as part of the SASP which increase the vulnerability to plaque rupture leading to cardiovascular complications. (Figure created with BioRender.com).

Senescent cells expressing SA‐β‐gal, p16^Ink4a^ and p21^Cip1^ have been identified within atherosclerotic plaques from human coronary artery samples [80, 81, 82]. A range of surface markers including endothelial cells [82], vascular smooth muscle cells (VSMC) [80] and infiltrating immune cells [73, 74] co‐stain with senescent markers, suggesting that a variety of cell types become senescent during atherosclerosis (Fig. 1). Atherosclerotic plaques most commonly develop at branch points of large coronary arteries where there are regions of low or disturbed flow, which coincides with the distribution of senescent cells in the vasculature [83]. The presence of senescent endothelial cells in these areas predates the development of atherosclerotic plaques and the number of senescent cells increases with the severity of the lesion [56, 74]. Disruption to laminar flow at arterial branch points exerts abnormal shear stress on the endothelial layer in this area which has been shown using *in vitro* models of disrupted laminar flow systems to induce a p53‐mediated senescence within human umbilical vein endothelial cells (HUVEC) [56]. Similar changes have been induced in HUVEC exposed to high levels of glucose within the culture media [59, 84] and following treatment with chemotherapeutic agents [53].

Senescent endothelial cells have a pro‐inflammatory SASP including TNF‐α, IL‐1, IL‐6 and IL‐8 [85, 86], which in combination with increased expression of vascular adhesion molecules on the luminal surface enhances recruitment of circulating immune cells to the vessel wall [82]. Macrophage infiltration into the vessel intima and phagocytosis of intimal cholesterol to form foam cells is an early important step in the development of atherosclerotic plaques and it is likely that the emergence of senescent cells in the endothelium contributes to or accelerates this process [87].

#### Senescent macrophages appear early in atherosclerosis and contribute to lesion progression

Foam cells are pathognomonic histological findings in atherosclerotic plaques and appear early during the development of lesions. During treatment with a high‐fat diet in transgenic hypercholerolaemic mice, foam cells started to appear as early as day 9 [74]. The majority of foam cells are formed by uptake of oxidized LDL by infiltrating macrophages and they are associated with increased expression of a range of pro‐atherosclerotic factors including vascular adhesion molecules, pro‐inflammatory cytokines and extracellular matrix remodelling enzymes [79]. Modified LDL has been suggested to have a causative role in the development of macrophage senescence as modified LDL within lysosomes increases lysosomal pH, inactivates proteases and lipases [88] and is not effectively degraded. Accumulation of modified lysosomal LDL increases oxidative stress within macrophages and induces a stress‐induced, p53‐mediated senescent phenotype including development of a pro‐inflammatory SASP [89].

#### Senescent VSMC impair plaque stability

Senescent VSMC within the plaque are of mesenchymal origin and contribute to ECM deposition within atherosclerotic plaques. ECM deposited within the plaque body increases plaque size and narrows the vessel lumen. ECM deposition at the fibrous cap reinforces the cap and reduces the risk of plaque rupture. Rupture of the atherosclerotic plaque results in acute occlusion of the vessel lumen leading to acute ischaemic events such as myocardial infarction or stroke and the stability of the fibrous cap is therefore an important contributor to disease progression [90]. VSMCs identified within the fibrous cap of atherosclerotic plaque have features of senescence including shortened telomeres with telomere length inversely correlated with disease severity. *In vitro* culture of plaque VSMC demonstrated reduced proliferation potential and increased expression of p16^Ink4a^, p21^Cip1^ and SA‐β‐gal compared to non‐plaque VSMC taken from the same individual [81]. Oxidative stress accelerates this process; recent evidence has highlighted downregulation of the histone deacetylase, Sirt6, in response to high‐fat diet and the saturated fatty acid palmitate as a key step in the development of VSMC senescence within atherosclerotic plaques linking this to metabolic syndrome [91]. The presence of VSMC in the fibrous cap of atherosclerotic plaques impaired ECM synthesis and increased expression of ECM remodelling enzymes such as the MMP with resulting thinning of the fibrous cap and increased susceptibility to plaque rupture [80, 92, 93].

#### Senolytic therapies in atherosclerosis

Given the abundance of evidence in multiple cell types suggesting a pro‐atherosclerotic role for senescent cells, selective targeting of these cells with senolytic compound holds promise in the prevention, regression and stabilization of atherosclerotic plaques. Roos et al have demonstrated that chronic treatment of aged mice with dasatinib and quercetin reduced the number of senescent cells within the vessel wall and improved measurements of endothelial function in aged mice. Although there was no change in the plaque size, there was a reduction in calcification of plaques, which has been shown to improve the efficacy of cholesterol‐lowering therapies [94]. Childs and colleagues employed both a genetic and pharmacological approach to senolysis in atherosclerosis using p16‐3MR mice and ABT263 respectively. They reduced the number of senescent endothelial cells, VSMC and macrophages in advanced lesions which was associated with reduction in plaque size. Clearance of senescent cells also improved the thickness of the fibrous cap by reducing expression of MMP, which reduces the risk of plaque rupture. Additionally, when applied to early lesions, clearance of senescent macrophages in fatty streaks leads to regression of plaque within the vessel [74].

However, potential questions remain regarding the safety of this approach. The role of senescence in the vasculature outside of atherosclerosis remains poorly understood and potential regulatory or homeostatic roles have not been excluded. Gross et al highlighted the potential pitfalls of this approach when using genetic approaches continuously eliminating p16‐high senescent cells. This approach targeted different senescent cells, including liver sinusoidal endothelial cells expressing high levels of p16^Ink4a^ [95]. Cleared senescent cells are not replaced adequately by non‐senescent endothelial cells resulting in increased vascular permeability in the liver and associated perivascular liver fibrosis. Whether these effects are associated with the continuous (rather than intermittent) elimination of senescent cells and whether specific senolytics differentially target distinct types of senescent cells needs further examination. Therefore, the long‐term effect of targeting senescent cells in the vasculature will need further study and optimization of both the duration and dose of therapy to prevent complications as seen in the Gross study. If these issues can be clarified, the potential to halt or induce regression of atherosclerosis if realized in the clinic would provide a paradigm shifting approach to primary and secondary prevention of cardiovascular disease.

### Myocardial infarction

One of the major complications resulting from atherosclerotic plaque rupture is an acute myocardial infarction (AMI). Obstruction of blood flow in a major epicardial coronary artery results in areas of acute ischaemia, in which highly metabolically active cardiomyocytes undergo rapid necrotic cell death. The size of the infarct depends on the area of myocardium, which is exclusively supplied by the affected coronary artery and the time before the occluded vessel is reopened, if at all. Restoration of blood flow to the area after a period of ischaemia also has consequences in the form of ischaemia‐reperfusion injury which is associated with a high degree of oxidative stress and cell damage. Cells in the peri‐infarct region or those exposed to the effects of ischaemia‐reperfusion experience high levels of stress and DNA damage. The response of these cells is key to defining the long‐term prognosis post‐infarct. The inflammatory and neurohormonal factors produced in these regions have the potential to exacerbate surrounding fibrosis, increase the infarct area and contribute to adverse remodelling of the remaining myocardium which have important prognostic implications [96, 97].

#### Senescent cells accumulate following AMI

AMI models *in vivo* have shown upregulation of p16^Ink4a^, p21^Cip1^, DNA damage markers and SA‐β‐gal expression within cardiomyocytes following 60 mins of ischaemia‐reperfusion or a completed infarct. SASP factors including IL‐1, IL‐6, TNF‐α and TGF‐β are also upregulated in both models [34, 37, 98, 99]. Analogous *in vitro* models involve culturing NRVCM at 1% oxygen to replicate a completed infarct or cycling between hypoxia and normoxia to replicate ischaemic reperfusion both of which induce cardiomyocyte senescence [99, 100, 101, 102].

Oxidative stress is thought to be key to this senescence response, with areas displaying the highest levels of oxidative stress post‐MI also producing the highest proportion of senescent cells [34]. Antioxidant therapies effectively reduce the accumulation of senescent cells within the myocardium post‐MI [100] with an associated reduction in infarct size, improvement of left ventricular function and reduced mortality [99]. Sirt‐1 is an NAD+‐dependent deacetylase which has a number of roles related to induction of senescence including deacetylation of p53 [103]. In ischaemic‐reperfusion models, upregulation of Sirt‐1 either genetically or pharmacologically has protective effects which reduce oxidative stress and reduce infarct size [104, 105]. In the aged heart, the nucleo‐cytoplasmic shuttling of Sirt‐1 is impaired, which reduces its activity leading to increased p53 activity and increased cardiomyocyte senescence [105].

#### Fibroblast senescence has fibrosis limiting effects following AMI

**Fig. 2:**
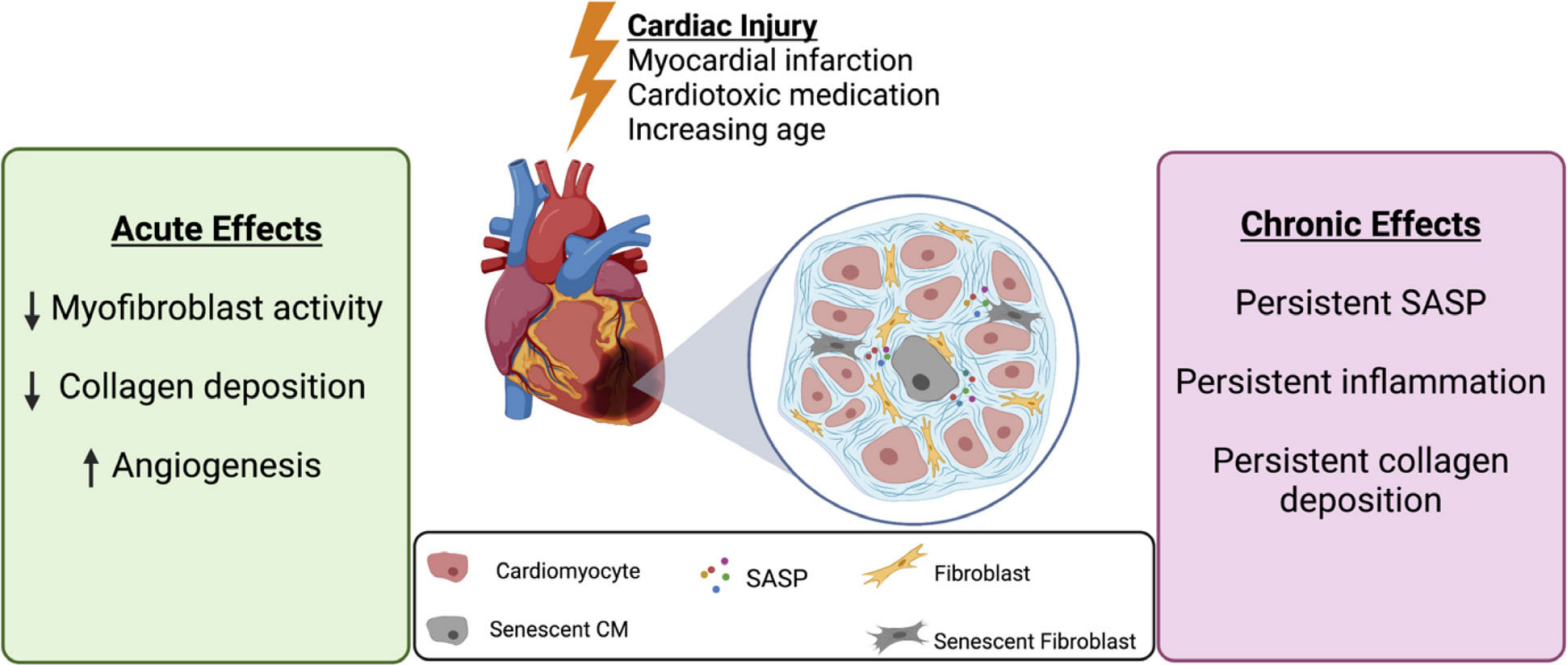
Acute and chronic effects associated with senescence induction during cardiac injury. Senescent cells accumulate within the ventricular myocardium in response to a range of stressors including myocardial infarction, cardiotoxic medication, systemic neurohormonal changes and increasing age. Senescence particularly in fibroblasts may have a short‐term role in limiting the excessive fibrotic response and improving early angiogenesis. In the chronic setting, senescent cells which are not effectively cleared by the immune system release a SASP which contributes to chronic inflammation, collagen deposition and development of myocardial hypertrophy. Targeting senescent cells in this chronic phase may reduce cardiac dysfunction following myocardial infarction and reduce hypertrophic changes associated with hypertension and ageing. (Figure created with BioRender.com).

Induction of senescence in cardiac fibroblasts in the acute phase following AMI is associated with some short‐term beneficial effects [106]. The rapid and coordinated activation of cardiac fibroblasts is crucial following AMI to replace and reinforce necrotic tissue with fibrotic tissue to provide structural support and prevent rupture of the ventricular wall which has catastrophic consequences (Fig. 2). However, achieving a balance to avoid overly aggressive inflammatory and fibrotic response is important to prevent excess ECM and immune cell recruitment which interferes with the function of the surviving myocardium and extends the infarct. The SA‐β‐gal‐positive cells which emerge in the heart up to 7 days post‐MI have been shown with co‐staining studies to predominantly express α‐SMA, suggesting that a large proportion of these cells are exhausted myofibroblasts [98]. In culture, hypoxia‐induced senescence in cardiac fibroblasts reduces expression of collagens and increases expression of ECM remodelling enzymes such as MMP [91]. In keeping with these findings, studies in mouse AMI models which circumvent senescence in fibroblasts by genetic manipulation of p53 or p53‐stabilizing proteins results in reduced accumulation of senescent cells but increased infarct size, increased fibrosis and increased mortality 28 days post‐MI. [37, 98, 106].

Senescent fibroblasts also stimulate angiogenesis in the ischaemic myocardium in the early period following AMI. Senescent endothelial cells increase expression of pro‐angiogenic factors such as vascular endothelial growth factor and promote the development of angiogenesis within the infarcted myocardium in mice [98].

Although these functions reflect a potentially important role of senescent cells in tissue homeostasis post‐AMI, the long‐term effects caused by large numbers of lingering senescence cells and their associated SASP have not been described in these studies. Additionally, when senescence is circumvented, fibroblasts with high levels of DNA damage are able to persist and replicate and may undergo necrotic cell death with associated inflammatory consequences. Although potentially adaptive in the short term to prevent excessive reparative fibrosis out of keeping with the extent of injury, the ongoing pro‐inflammatory SASP from these cells over the long term is likely to result in chronic collagen deposition and heart failure [37].

#### Endothelial senescence increases the risk of coronary events

The response to myocardial infarction and in particular ischaemia‐reperfusion injury includes high levels of oxidative stress and age is an independent predictor of death despite adjustment for co‐morbidities [3]. One explanation for this is that increased number of accumulated senescent cells in older patients exacerbate the response to injury and prevent effective regeneration. Expression of SA‐β‐gal, p16^Ink4a^, p21^Cip1^ and p53 increases with passage of primary endothelial cells. These cells have reduced nitric oxide synthase activity and their ability to inhibit platelet aggregation is impaired which predisposed to coronary events [64]. Microparticles present in the plasma of patients following acute coronary syndrome have been shown to induce premature senescence in endothelial cells. These changes appear to be dependent on angiotensin II, which mediates and increases on NAD(P)H oxidase activity [64].

Circulating endothelial progenitor cells (EPC) are haematopoietic cells which have been reported to be involved in repair of vascular endothelial injury most likely through paracrine mechanism rather than directly incorporating in the vessel [107]. Circulating endothelial cells from older individuals demonstrate increase in SA‐β‐gal activity and reduced proliferative capacity [108]. Depletion of TERT or Gdf11 results in senescence in young EPC leading to impaired angiogenesis. When used to augment myocardial repair post‐infarction, the beneficial effects of EPC on cardiac function and the survival benefit are lost in cells treated with Gdf11 or TERT inhibitors [108]. Clinically, senescence in this population of cells correlates with risk of restenosis of coronary stents following percutaneous coronary intervention, suggesting an inability to positively remodel the vasculature [109].

#### Senolytics post‐AMI

Given the early potentially beneficial effects of senescence cells, the use of senolytic therapies in this sphere requires some caution. Particular attention should be paid to the timing of therapy to avoid eliminating the potentially beneficial early effects of senescent cells. Limited studies have been conducted to date investigating senolytic therapies post‐MI. The BCL‐2 inhibitor ABT263 has been used in mice by Dookun and colleagues following a 1‐h ischaemia‐reperfusion protocol. Treatment with ABT263 was started on the 4^th^‐day post‐AMI and given daily for 7 days. This delayed administration of senolytic therapy allows early reparative fibrosis to be established and follows a switch from TGF‐β1 which promotes reparative fibrosis to TGF‐β3 which is associated with interstitial fibrosis. They found that ABT263 therapy improved myocardial remodelling, left ventricular function and reduced mortality post‐MI. The SASP was reduced with less pro‐fibrotic and pro‐inflammatory signalling [34].

Interestingly, senolysis also appeared to improve angiogenesis within the infarct region, which the authors speculate may be attributable to a reduction in anti‐angiogenic SASP components such as IP‐10. However, endothelial senescence limits angiogenesis and direct senolytic effects on the endothelial cells also warrant mechanistic consideration. This study does not identify the specific cell types targeted by senolysis. It is likely that clearance of a combination of cell types will be responsible for the beneficial effects and if proved safe may be a crucial treatment to prevent worsening of cardiac dysfunction developing post‐AMI. A second study from this group pre‐treated aged animals with ABT263 prior to myocardial infarction and found that cardiac function and survival were improved following AMI [110]. These results indicate that senescent cells within the myocardium accumulate with age and may be a significant contributor towards the increased mortality seen with myocardial infarction at advanced age. Although it is unclear what cell type is targeted, this study provides important insights into the role that senescence plays in cardiovascular morbidity.

### Heart failure

Heart failure is a clinical syndrome characterized by the inability of the heart to generate sufficient cardiac output to meet the physiological needs of the body. This can be a consequence of another pathology such as AMI, hypertension or myocarditis or can be a primary cardiac defect such as dilated or hypertrophic cardiomyopathies. The haemodynamic effects can be due to impairment in the contractile function due to the loss of force‐generating cardiomyocytes, disruption of myocyte architecture due to fibrosis, impairment in the myocardial compliance during diastole‐limiting myocardial filling or a combination of these. Heart failure is increasingly common with age and has a high associated morbidity and mortality [111].

#### Neurohormonal activation in heart failure induces senescence within the heart

The activation of the sympathetic nervous system and renin–angiotensin–aldosterone system occurs in the pathogenesis of heart failure and is responsible for perpetuating adverse cardiac remodelling, increasing cardiac fibrosis and cardiac hypertrophy. β‐adrenergic receptor antagonists and inhibitors of the RAAS are the cornerstone of modern heart failure therapy and have resulted in a remarkable improvement in survival over recent decades [112, 113]. Commonly used pharmacological mouse models of heart failure include angiotensin II infusion and low‐dose infusion of the β‐adrenoceptor agonist isoproterenol. Both of these models induce p16^Ink4a^ and SA‐β‐gal expression in cardiomyocytes and fibroblasts in the myocardium. A similar increase in senescent cells has been identified using transverse aortic constriction, a mechanical pressure overload model of heart failure. However, in this study, co‐staining experiments revealed that the majority of p21^Cip1^‐positive cells co‐stain for platelet‐derived growth factor receptor and α‐smooth muscle actin (α‐SMA) suggesting a myofibroblasts origin. Only a very small proportion of cells are CD31 or cardiac troponin T positive indicating minimal contribution of endothelial or cardiomyocyte, respectively, to the senescent population [41]. Similarly, overexpression of monoamine oxidase A (to cause oxidative stress) using a cardiomyocyte‐specific promoter resulted in systolic dysfunction. Mesenchymal stromal cells (MSC) were the primary cell developing senescence and the degree of MSC senescence negatively correlated with systolic function [114]. Cardiomyocytes extracted from aged mouse heart demonstrate similar features to neurohormonal and pressure overload models of heart failure with features of senescence including SA‐β‐gal, p16^Ink4a^ and p21^Cip1^expression and expression of prohypertrophic genes [20].

#### Fibroblast senescence in heart failure may limit fibrosis, however, is pro‐inflammatory

As discussed, senescent cardiomyocytes display an atypical SASP. Conditioned media from senescent cardiomyocytes cause fibroblast‐to‐myofibroblast activation and the induction of senescence in neonatal fibroblasts [20]. Studies treating cardiac fibroblasts *in vitro* with the saturated fatty acid palmitate have shown that 72 h following stimulation fibroblasts develop endoplasmic reticulum stress, reduced proliferative potential and expression of SA‐β‐gal. As described in AMI models, fibroblast senescence is associated with reduced collagen expression from senescent cells, however, upregulation of the pro‐inflammatory secretome which would be expected to cause progressive tissue damage over time [98]. Sawaki and colleagues have demonstrated that osteopontin, an adipokine which is released from adipose tissue, prevents the induction of senescence in cardiac fibroblasts and promotes worsening of fibrosis within the heart and reduced myocardial function compared to animals where osteopontin is knocked out [115]. The authors suggest that by preventing induction of fibroblast senescence in obesity, there is exaggerated fibrotic response within the myocardium and impairs cardiac haemodynamics [115].

Capillary endothelial cells within the microvasculature of the coronary circulation provide important signalling functions within the heart which have been linked to heart failure. In particular, deficiencies in nitric oxide signalling have been extensively described in the pathogenesis of heart failure with preserved ejection fraction [47]. Studies using senescence accelerated mice fed a high‐fat, high‐sugar western diet for 16 weeks develop increased SA‐β‐gal expression within the endothelium and have reduced expression of endothelial nitric oxide synthase (eNOS). Acetylcholine‐induced vasodilation, which is an endothelium‐dependent process, is impaired and these mice show evidence of diastolic impairment on echocardiography [102].

#### Effects of preventing senescence are variable between studies

The cellular identity of senescent cells and the effects of limiting senescence are not consistent between studies and may depend upon the specific model used or the mechanism for avoiding senescence. Resveratrol is a treatment which prevents the onset of senescence through activation of Sirt‐1 and AMPK, thereby improving autophagy, improving mitochondrial biogenesis, reducing oxidative stress and improving lifespan in mice [116]. Co‐administration of resveratrol with isoproterenol reduced the accumulation of senescent cardiomyocytes and reduced cardiomyocyte hypertrophy and natriuretic peptide expression [117]. In contrast, genetic knockout of p16^Ink4a^ and p53 in pressure overload mouse models prevented the onset of senescence in cardiac fibroblasts and resulted in increased perivascular fibrosis and reduced left ventricular function compared to wild‐type animals [41]. This apparent difference in effect of the respective senescence limiting treatments may be related to the stage of cell damage when these effects become relevant. Knockout of p16^Ink4a^ and p53 will preserve the function and proliferative potential of fibroblasts containing high degree of cellular damage which may exacerbate ongoing cardiac injury. Upstream targets which prevent or improve cellular damage such as improving autophagy or reducing oxidative stress may be able to prevent the accumulation of cell damage and tissue dysfunction therefore providing more beneficial therapeutic effects.

#### Senolytics in heart failure

Limited trials of senolytics in mouse models of heart failure have been undertaken. Senolytic therapy using either genetic or pharmacological methods reduces cardiomyocyte hypertrophy in aged animals [20, 118]. Remarkably, short‐term 2‐week treatment with ABT263 in 2‐year‐old mice just prior to sacrifice has shown a significant improvement in cardiac fibrosis and cardiomyocyte hypertrophy which had developed chronically over 2 years of normal ageing [20].

Senolytic therapy reduces the adverse remodelling associated with neurohormonal activation in heart failure. Clearance of p16^Ink4a^‐positive cells in the INK‐ATTAC mouse prevents the increase in left ventricular mass which occurs in response to low‐dose β‐adrenergic stimulation with isoproterenol. ABT263 reduces the number of senescent fibroblasts and cardiomyocytes due to AngII infusion which reduced cardiac fibrosis and cardiomyocyte hypertrophy and improved systolic function. [119]. Senolysis also improved the inflammatory state of the myocardium with reduced expression of SASP factors, IL‐6 and TNF‐α, reduced expression of vascular adhesion molecules and reduced myocardial immune cells infiltration.

Dasatanib and quercetin in combination have been demonstrated to have senolytic properties on various cell types including endothelial cells. Quercetin was used in high‐fat diet‐treated mice and limited the reduction in myocardial capillary density which is a feature of heart failure and improved tube‐forming ability of HUVEC challenged with oxidized LDL [62]. Quercetin alone did not show senolytic activity *in vivo,* however, with the addition of dasatinib there was a significant senolytic effect *in vitro* on irradiated senescent HUVEC [12]. A single dose of dasatinib and quercetin administered to a 24‐month‐old mouse improved both endothelial‐dependent and ‐independent vasomotor function of the carotid artery [12] and in the heart improved left ventricular systolic function and reduced the accumulation of fibrosis [12, 77].

However, despite these promising initial results, many unanswered questions remain regarding the function and role of senescent cardiomyocytes within the myocardium. Some studies show a high proportion of cardiomyocytes within the myocardium are senescent and the loss of these cells through the induction of apoptosis by senolytic therapy would be expected to have significant impact on systolic function. Although some evidence exists to suggest that cardiac progenitor cells activity is increased by senolytic therapy, it is unlikely this will be sufficient alone to replace the large number of senescent cells which are reportedly being cleared in these studies. Although no impairment in cardiac function was noted immediately following senolytic treatment, further longer‐term studies will be needed to ensure this persists over longer time periods.

### Cardio‐oncology

Cardiotoxicity following cancer therapy is a growing problem as cancer survivorship improves and patients cured of cancer develop late‐onset heart failure due to cardiotoxicity of the treatments. Anthracycline‐induced cardiotoxicity has been the most commonly studied and is particularly problematic in survivors of paediatric cancers where doxorubicin remains a first line, highly effective therapy and the timeframe for the development of side‐effects is longer. Up to 25% of doxorubicin‐treated patients develop some degree of cardiotoxicity with increasing incidence correlating with the cumulative lifetime dose [120]. The potential for these chemotherapeutic agents to induce senescence within the cardiovascular system and the effect this may have on long‐term cardiac performance and resilience to future cardiac insults has become an active area of investigations in recent years.

#### Anthracyclines cause accumulation of senescent cells

*In vitro* evidence points to a pro‐senescent effect of anthracyclines in cardiomyocytes with increased SA‐β‐gal expression, telomere shortening, upregulation of p53 and p16^Ink4a^ expression and damage to mitochondrial DNA being demonstrated in NRVCM and H92c cells following doxorubicin treatment [121, 122, 123, 124, 125]. High‐dose doxorubicin results in widespread apoptosis in cardiomyocytes, however, low‐dose doxorubicin downregulated telomere‐binding protein, TRF2, causing p38‐mediated senescence in these cells [126]. These senescent cells are vulnerable to further cancer therapies targeting pro‐survival pathways such as the erbB2 receptor [127]. This has important clinical implications due to multi‐targeted chemotherapy regimens such as the addition of trastuzumab in breast cancer chemotherapy regime which causes apoptosis in these cells *in vitro* and is associated with high rates of cardiotoxicity in clinical practice [128].

Anthracycline chemotherapy along with a number of other commonly used chemotherapeutic agents from different classes [53, 129] cause endothelial senescence in culture, which may contribute to the cardiotoxicity profile *in vivo*. Doxorubicin‐treated endothelial cells express SA‐β‐gal, p16^Ink4a^ and p21^Cip1^ and develop a flattened morphology characteristic of senescent cells [130]. Functionally these cells have reduced tube‐forming ability on matrigel [131] and reduced expression of vasoactive mediators such as nitric oxide [132], suggesting impairment in the vasoactive and angiogenic functions of EC in senescence. *In vivo* studies support these findings as capillary density is reduced in doxorubicin‐treated mice and vascular permeability is increased [133, 134]. Mice treated with doxorubicin in the first 3 weeks of life have reduced myocardial vascularity as adults and develop myocardial hypertrophy and LV dilation when required to exercise in adulthood [135]. These mice were more susceptible to myocardial infarction with a higher mortality and reduced neovascularization of the border zone [135]. Circulating endothelial progenitor cells which play a role in cardiac angiogenesis have also been shown to undergo senescence in humans after doxorubicin treatment limiting their angiogenic potential [127]. This suggests that doxorubicin‐induced senescence of endothelial cells may prevent effective neovascularization in response to myocardial hypertrophy or increased myocardial oxygen demand to respond to stressful stimuli in adulthood. This is particularly relevant given that doxorubicin remains first line in many childhood cancers and senescence‐inducing treatments early in life may render the person more vulnerable to common cardiac insults in the future such as hypertension, myocardial infarction or secondary cardiotoxic agents. Care to manage cardiovascular risk factors in this group is likely to be of even greater importance as there is likely to be reduced physiological reserve compared to their peers.

#### Current licenced cardiac oncology treatments have anti‐senescence effects

Currently, the only drug licenced for the prevention of anthracycline cardiotoxicity is the chelating agent dexrazoxane. Dexrazoxane reduces oxidative stress within the cell and cotreatment of H9c2 cells, and NRVCM *in vitro* with dexrazoxane reduces the pro‐senescence effects of anthracyclines and avoids sensitizing these cells to erbB antagonists [121]. Recently, alternative preventative therapies have been proposed for anthracycline cardiotoxicity both of which target PAI‐1 pro‐senescence pathway [136]. A small molecule inhibitor of PAI‐1 reduces doxorubicin‐mediated senescence in multiple cells types including endothelial cell, fibroblasts and cardiac myofibroblasts as well as reducing oxidative stress within endothelial cells [130]. Roflumilast is a phosphodiesterase‐4 inhibitor which prevents doxorubicin‐induced downregulation of Sirt1 and upregulation of PAI‐1 in H9c2 cells and prevents onset of senescence [122]. Similarly, administration of a combination of resveratrol and quercetin in combination to H9c2 cells reduced cell death in these cells which was thought to be related to increased scavenging of ROS [137]. Quercetin when added to the angiotensin receptor blocker losartan is able to reduce pro‐inflammatory SASP components such as TNF‐α, reduce oxidative stress, reduce myocardial infiltration with immune cells and lead to improved cardiomyocyte survival [138].

#### Senolytics in cardio‐oncology

Doxorubicin therapy induces significant senescence in the myocardium which render the heart vulnerable to secondary insults or increased workload during stress. Control of secondary insults following cardiotoxic chemotherapy is likely to be of paramount importance and control of cardiovascular risk factors should take an even greater significance in these patients who appear to have a lifelong intolerance to cardiac stressors. Recent studies have demonstrated a potential role for senolytic treatments in this arena. Demaria et al have used the p16‐3MR mouse to demonstrate beneficial effects of clearing senescent cells following doxorubicin therapy. They identified the majority of senescent cells to be either CD31‐positive endothelial cells or fibroblasts rather than cardiomyocytes, and the clearance of these cells resulted in improvement in cardiac function [139]. However, other studies indirectly raise potential concerns of senolytic therapies in cardiovascular disease. Spallarosa and colleagues' study demonstrated that pro‐senescent doses of anthracycline in combination with erbB1 antagonists resulted in significant apoptosis of cardiomyocytes effectively acting as a senolytic therapy [127]. However, this combination of anthracyclines and trastuzumab has significant cardiotoxic effects and understanding of the contribution of apoptosis of senescent cardiomyocytes to this toxicity is important before proceeding with clinical trials in this setting.

### Pulmonary hypertension

Pulmonary hypertension (PH) involves the progressive obstruction of the pulmonary vasculature, reducing the ability for gas exchange and increasing pressures within the pulmonary circulation and right heart. PH has a wide range of causes including primary inherited conditions as well as secondary to pro‐inflammatory, autoimmune conditions or those affecting the loading conditions with the vessel such as congenital heart disease [140]. Similar to atherosclerotic models, the presence of senescent cells has been demonstrated within all three layers of the pulmonary arterial vessel wall in mouse models of PH [141, 142, 143].

As has been seen in the setting of AMI, there is evidence that senescence of secretory fibroblasts in the acute phase of PH may be beneficial to prevent excessive hyperplasia and fibrosis accumulation. Stabilizing p53 in the setting of hypoxia‐induced senescence in pulmonary artery VSMC reduced muscularization of the pulmonary vessels and improved right heart haemodynamics compared to controls [144]. However, the persistence of senescent cells beyond this acute phase within the pulmonary vasculature has been associated with development of progressive and irreversible vascular changes even if normal haemodynamics can be restored [143]. Genetic factors which predispose to mitochondrial dysfunction and ROS formation within pulmonary endothelial cells have been identified as key mediators of senescence development and subsequent progression of PH [145, 146, 147]. Additionally, *in vitro* studies with cells isolated from the pulmonary microvasculature have indicated that abnormal shear stress plays a role in the development of senescence which was more marked in cells from patients with PH than control donors [143]. In models of PH associated with congenital heart disease, where pulmonary haemodynamics are typically highly abnormal, the abnormal flows that exist and provide a positive feedback loop to perpetuate the damage as the vascular haemodynamics become increasingly abnormal. The SASP associated with pulmonary endothelial senescence includes TNF‐α and IL‐6 and is capable of stimulating VSMC activation and pathological vessel remodelling [143]. Abnormalities in nitric oxide signalling is a key step in the development of PH and its release is impaired in senescent pulmonary artery endothelial cells. Endothelial nitric oxide synthase has been shown to become trapped within the endoplasmic reticulum and not trafficked to its site of action in the calveoli leading to reduced NO production [148].

#### Senolytics in PH

Recently, a number of trials undertaken using ABT263 in murine PH models have provided promising results. Although the cellular target of these therapies requires further confirmation, it appears that senescent cells within both the endothelial and VSMC populations are targeted. The SASP is downregulated and recruitment of immune cells to the pulmonary vasculature is reduced which is able to prevent or reverse the histological changes within pulmonary arteries. As a result, ABT263 has improved the haemodynamics in pulmonary circulation and the right heart [143, 145] and rendered previously irreversible PH amenable to treatment. Given that the elimination of endothelial cells has been shown to produce vascular leakage in other contexts [95], the right senolytics (targeting specific senescent subpopulations) and appropriate dosage (regulating the duration of the therapies) will be needed to reach a therapeutic window effective for PH treatment.

## Conclusion

Increasingly, evidence has suggested a contribution of senescent cells in the cardiovascular system in the pathophysiology of disease and in particular chronic cardiovascular diseases such as atherosclerosis and heart failure. Preclinical studies have repeatedly demonstrated a beneficial effect associated with the use of senolytic therapies and the clearance of senescent cells from cardiovascular organs. The precise contribution of various cell types to the senescent population within the heart and cardiovascular organs requires further definition and alternative theories exist regarding the predominant cellular target providing the beneficial effects with senolytic therapy. Ongoing single‐cell studies will serve to identify the nature of the senescent cells contributing to cardiac disease. Additionally, as is the case in malignancy, cellular senescence has important and necessary effects in the cardiovascular system. These have been demonstrated most clearly with fibroblasts where the avoidance of senescence leads to excessive fibrosis accumulation in response to pathological stimuli. However, a similar problem does not appear to emerge with senolytic clearance of fibroblasts, suggesting senescence is a necessary short‐term homeostatic process in pro‐fibrotic heart; however, persistence of these non‐secretory pro‐inflammatory cells induces long‐term ongoing tissue damage which may prevent senolytic clearance. The fact that clearance of senescent cells rapidly reverses long‐standing fibrosis and hypertrophy suggests that these cells have an active role perpetuating tissue damage in both diseased and aged myocardium.

The finding that adult cardiomyocytes undergo senescent changes and are susceptible to senolytic therapies highlights an intriguing opportunity to treat age‐related heart failure. However, this also raises important questions regarding the contractile function of senescent cardiomyocytes. Targeted clearance of these cells particularly in the adult heart, where the ability to regenerate cardiomyocyte is limited, may have significant implications for long‐term cardiac function. Moreover, clarification of these findings and their implications will be important to reassure regarding the long‐term safety of senolytic drugs in any setting, even outside cardiovascular system.

Caution with dosing regimens and careful attention to timing of intervention are required to address these safety concerns. Additionally, close attention must be paid to off‐target toxicities from senolytic therapies in non‐malignant disease as toxicities which are routinely tolerated in chemotherapy regimens would be dose limiting or intolerable, particularly when used in preventative cardiology. However, these challenges are unlikely to be insurmountable obstacles to bringing senolytic therapy in the cardiovascular system to clinical trials. A better understanding of the senescent cells mediating cardiac disease will allow senolytic therapies to be tailored to maximize effectiveness while avoiding side effects. Ongoing trials in non‐cardiac organs have already demonstrated a degree of safety with senolytic therapy and promising results from these trials will provide further evidence to support the application of these therapies to high prevalence cardiovascular disease where they could have wide‐reaching implications for managing these chronic age‐related diseases.

## Conflict of interest

JG has acted as a consultant for Unity Biotechnology, Geras Bio, Myricx and Merck KGaA. JG owns equity in Geras Bio. JG is a named inventor in MRC and Imperial College patents related to senolytic therapies. SAC is a co‐inventor of the patent applications (WO/2017/103108: TREATMENT OF FIBROSIS, WO/2018/109174: IL‐11 ANTIBODIES, WO/2018/109170: IL‐11RA ANTIBODIES). SAC is a co‐founder and shareholder of Enleofen Bio PTE LTD, a company that develops anti‐IL‐11 therapeutics.

## Author contributions

JG, SC and MS conceived the review. MS wrote the original draft. JG, SC and MS reviewed and edited the manuscript.
